# T cells bearing anti-CD19 and/or anti-CD38 chimeric antigen receptors effectively abrogate primary double-hit lymphoma cells

**DOI:** 10.1186/s13045-017-0488-x

**Published:** 2017-06-08

**Authors:** Keichiro Mihara, Tetsumi Yoshida, Yoshifumi Takei, Naomi Sasaki, Yoshihiro Takihara, Junya Kuroda, Tatsuo Ichinohe

**Affiliations:** 1 Department of Hematology and Oncology, Research Institute for Radiation Biology and Medicine, Hiroshima University, 1-2-3 Kasumi, Minami-ku, Hiroshima 734-8553 Japan; 2 Department of Medicinal Biochemistry, School of Pharmacy, Aichi Gakuin University, Nagoya, 470-0195 Japan; 3 Department of Pathology, Kure Kyosai Hospital, Kure, 737-0811 Japan; 4 Department of Stem Cell Biology, Research Institute for Radiation Biology and Medicine, Hiroshima University, Hiroshima, 734-8553 Japan; 5 Division of Hematology and Oncology, Kyoto Prefectural University of Medicine, Kyoto, 602-8566 Japan

**Keywords:** T cell immunotherapy, Double-hit lymphoma, Double-expressing lymphoma, Chimeric antigen receptor (CAR), Anti-CD19-CAR, Anti-CD38-CAR

## Abstract

Patients with B cell lymphomas bearing *MYC* translocation combined with translocation involving other genes, such as *BCL2*, *BCL3*, or *BCL6*, defined as double-hit lymphoma (DHL), have a poor prognosis. Recent studies expanded the concept to include double-expressing lymphoma (DEL) that co-overexpresses MYC protein with either of those proteins. Accordingly, we defined cytogenetic DHL and DEL as primary DHL. An adoptive T cell immunotherapy with a chimeric antigen receptor (CAR) has been clinically shown to exhibit cytotoxicity in refractory neoplasias. We revealed the marked cytotoxicity of anti-CD19- and/or anti-CD38-CAR T cells against primary DHL cells from patients. CD19- and/or CD38-specific T cells were co-cultured with cytogenetic DHL (*n* = 3) or DEL (*n* = 2) cells from five patients for 3 days. We examined whether T cells retrovirally transduced with each vector showed cytotoxicity against DHL cells. Anti-CD19- and/or anti-CD38-CAR T cells were co-cultured with primary DHL cells at an E:T ratio of 1:2 for 3 days. Anti-CD19- and anti-CD38-CAR T cells completely abrogated these DHL cells, respectively. Anti-CD19-CAR T cells synergistically exerted collaborative cytotoxicity against these primary DHL cells with anti-CD38-CAR T cells. Therefore, refractory DHL cells can be efficiently abrogated by the clinical use of T cells with anti-CD19- and/or anti-CD38-CAR.

**Electronic supplementary material:**

The online version of this article (doi:10.1186/s13045-017-0488-x) contains supplementary material, which is available to authorized users.

## Letter to the Editor

Patients with B cell lymphoma bearing *MYC* translocation combined with an additional translocation involving other genes, such as *BCL2*, *BCL3*, *BCL6*, or *CCND1*, whose category is defined as double-hit lymphoma (DHL), have a dismal prognosis [1]. Li et al. reported that the prognosis of B cell lymphoma patients expressing concurrent MYC and BCL2 proteins without translocations was also dismal as well as that of DHL-bearing translocations of *MYC/BCL2* genes in terms of the prognosis [1–4]. Thus, recent studies have expanded the concept to include double-expressing lymphoma (DEL) that co-overexpresses MYC protein with those proteins. Accordingly, we defined cytogenetic DHL and DEL as primary DHL. An adoptive T cell immunotherapy with anti-CD19 chimeric antigen receptors (CAR) has been clinically shown to exhibit marked cytotoxicity in patients with relapsed and refractory B cell lymphoid neoplasias [5–7]. We also developed anti-CD38-CAR and demonstrated its marked cytotoxicity against various hematological malignancies [8, 9]. However, it has not been elucidated whether CAR therapy could be effective for patients with cytogenetic DHL and DEL. Here, we revealed the marked cytotoxicity of anti-CD19- and/or anti-CD38-CAR T cells as well as the synergy of both CARs against primary DHL cells.

Cytogenetic DHL (*n* = 3) or DEL (*n* = 2) cells of the lymph nodes were collected from five patients (Table 1) after obtaining appropriate informed consent. CD19^+^ CD20^+^ lymphoma cells accounted for over 90% (90–97%). DHL (DEL) cell line cells, bearing the translocation of the *IgH/MYC* gene as well as overexpression of BCL2 protein (KPUM-UH1) or these primary cells were cultured in RPMI-1640 complete medium.

**Table 1:** Patients’ profiles and cytotoxicity of T cells expressing anti-CD19- or anti-CD38-CAR against primary DHL cells

Cells	Karyotype (major abnormalities)	IHC-positive	FISH-positive	^a^Expression of CD38 in CD19^+^ cells (%)	^a^Specific cytotoxicity of anti-CD19-CAR T cells (%)	^a^Specific cytotoxicity of anti-CD38-CAR T cells (%)
Patient 1	t(8;22)(q24;q11.2),t(14;18)(q32;q21)	BCL2 MYC: ND	BCL2 MYC	98.69 ± 0.37	92.67 ± 0.55	97.49 ± 0.19
Patient 2	add(8)(q24),t(8;14)(q24;q32), t(3;22)(q27;q11.2)	BCL2 BCL6 MYC	BCL6 MYC	98.74 ± 0.59	95.53 ± 2.88	99.88 ± 0.73
Patient 3	ND	BCL2 BCL6 MYC	BCL6	97.37 ± 0.02	98.94 ± 0.03	99.60 ± 0.27
Patient 4	ND	BCL2 MYC: ND	BCL2 MYC	98.47 ± 0.26	98.26 ± 0.78	99.47 ± 0.04
Patient 5	+8,add(3)(q27)	BCL2 BCL6 MYC	BCL6	97.14 ± 0.83	97.41 ± 0.16	97.70 ± 0.23

Specific cytotoxicity was evaluated by flow cytometry following the co-incubation of T cells bearing anti-CD19- or anti-CD38-CAR (E) with DHL cells (T) at an E:T ratio of 1:2 for 3 days. The cutoffs for positivity for BCL2, BCL6, or MYC were 50, 30, and 40% of the cells, respectively

*ND* not determined

^a^Results are the mean ± SD of three experiments

The cutoffs for immunohistochemical positivity for BCL2, BCL6, and MYC (Abcam, Cambridge, MA, USA) were 50, 30, and 40% of microscopically observed lymphoma cells, respectively. FISH analyses were performed by SRL (Tokyo, Japan).

The retroviral vector of anti-CD19- and anti-CD38-CAR was previously developed [8–10]. To produce a RD114-pseudotyped retrovirus, MSCV-IRES-EGFP-anti-CD19-CAR or MSCV-IRES-EGFP-anti-CD38-CAR, pEQ-PAM3(-E), and pRDF were used to co-transfect 293T cells with Lipofectamine plus (Invitrogen, Carlsbad, CA, USA). Peripheral blood mononuclear cells of donors were cultured for 48 h with 7 μg/ml PHA-M (Sigma, St Louis, MO, USA), 200 IU/ml interleukin-2 (PeproTech, London, UK) in the complete medium as described previously [8–10]. These T cells were retrovirally transduced in the presence of 4 μg/ml polybrene (Sigma) in a retronectin-coated tube (Takara-Bio, Otsu, Japan). For the transduction of anti-CD38-CAR, an anti-CD38 antibody (CPK-H; MBL, Nagoya, Japan) was added to the culture medium to protect transduced T cells from autolysis through cross-linkage of the anti-CD38-CAR with intrinsic CD38 [8, 9]. For the subsequent co-culture experiments, transduced T cells expressing green fluorescent protein (GFP) were sorted by FACSAria (BD). The specimens from patients and donors were used after approval by the institutional review board of Hiroshima University.

Primary DHL cells co-cultured with anti-CD19- and/or anti-CD38-CAR T cells were harvested and stained with an anti-CD19 antibody-PE and anti-CD38 antibody-APC (BD). These cells were then analyzed by a flow cytometer. Specific cytotoxicity of anti-CD19- and/or anti-CD38-CAR T cells against CD19^+^ primary DHL cells was evaluated using the formula (B-A)/B, where A is the number of CD19^+^ GFP^−^ cells or CD38^+^ GFP^−^ cells after incubation with anti-CD19- or anti-CD38-CAR-expressing T cells, respectively, and B is the number of CD19^+^ GFP^−^ or CD38^+^ GFP^−^ cells after incubation with vector-transduced T cells [8–10].

We initially detected cytogenetic DHL and DEL (Additional file 1: Figure S1 and Table 1). Next, we confirmed that goat anti-mouse-IgG-PerCP, which cross-reacts with CAR and GFP of the vector, were co-expressed as an internal control in T cells retrovirally transduced (transduction efficiency: 67.42 ± 14.43% (*n* = 5) for anti-CD19-CAR, 63.21 ± 10.89% (*n* = 5) for anti-CD38-CAR).

**Fig. 1:**
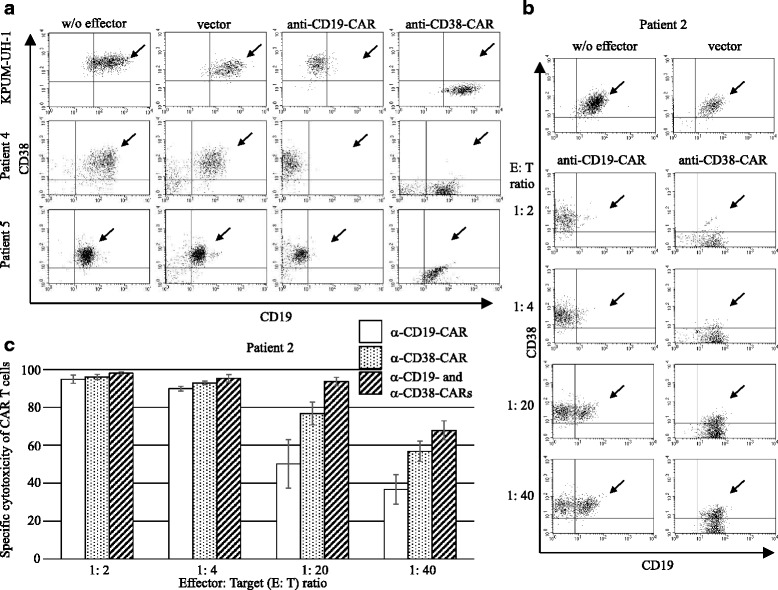
Cytotoxic effect of T cells with anti-CD19- and/or anti-CD38-CAR against DHL cells. **a** KPUM-UH1(DHL cell line) cells were co-cultured with mock, anti-CD19-, or anti-CD38-CAR T cells at an E:T ratio of 1:2 for 3 days. The cells were harvested and stained with an anti-CD38 antibody-APC and anti-CD19 antibody-PE. These cells were then analyzed by a flow cytometer. Anti-CD19- or anti-CD38-CAR T cells killed KPUM-UH1 cells, respectively (*upper panels*). Primary DHL cells from patients (patients 4 (cytogenetic DHL) and 5 (DEL)) were co-cultured with either of mock, anti-CD19-, or anti-CD38-CAR T cells at an E:T ratio of 1:2 for 3 days. Anti-CD19- or anti-CD38-CAR T cells eliminated primary DHL cells, respectively (*middle* and *lower panels*). The viable primary DHL cell population is indicated by the *arrowhead*. **b** Cytogenetic DHL cells from patient 2 (1 × 10^5^ cells) were co-cultured with anti-CD19- or anti-CD38-CAR T cells for 3 days at various ratios to effector cells (0.5 × 10^5^, 0.25 × 10^5^, 0.05 × 10^5^, and 0.025 × 10^5^ cells). Each type of CAR T cells abrogated cytogenetic DHL cells in a cell-number-dependent manner. The viable cytogenetic DHL cell population is indicated by the *arrowhead*. **c** The specific cytotoxic effect of anti-CD19- and/or anti-CD38-CAR transduced T cells against DHL cells was cell-number-dependent

Prior to co-culture experiments, we examined whether CD19^+^ primary DHL cells expressed CD38. We showed that >97% of DHL cells obtained from five patients expressed CD38 (Table 1). DHL (DEL) cell line cells (KPUM-UH1) expressing CD19 and CD38 were co-cultured with anti-CD19- or anti-CD38-CAR T cells at an effector (E) target (T) ratio of 1:2 for 3 days. Co-culture experiments showed that either anti-CD19- or anti-CD38-CAR T cells almost completely eradicated KPUM-UH1 cells (Fig. 1a). As further experiments, CD19- or CD38-specific T cells were co-cultured with cytogenetic DHL (*n* = 3) or DEL (*n* = 2) cells from five patients at an E:T ratio of 1:2 for 3 days. Similarly, anti-CD19- or anti-CD38-CAR T cells completely abolished the primary DHL cells, respectively (Fig. 1a, c and Table 1). Using DHL cells from patient 2, we confirmed that each of the CAR T cells eliminated DHL cells in a cell-number-dependent manner (Fig. 1b, c). Additionally, anti-CD19-CAR T cells synergistically exerted a collaborative cytotoxicity against DHL cells from patient 2 with anti-CD38-CAR T cells, as shown in Fig. 1c. The simultaneous combination index was less than 1.0, leading to the synergy according to Calcusyn software (Biosoft, Cambridge, UK).

These results showed that primary DHL cells, which are refractory or resistant to existing chemotherapeutic agents, can be efficiently abrogated by the clinical use of T cells with anti-CD19- and/or anti-CD38-CAR. Taken together, these results may warrant adoptive immunotherapy with T cells transduced with anti-CD19- and/or anti-CD38-CAR for patients with refractory cytogenetic DHL and DEL.

## Additional files

Additional file 1: Figure S1. Morphology of cells in the specimens on hematoxylin-eosin staining is shown. MYC expression is shown in lymph node specimens from patient 3. LPF, MPF, and HPF denote low-power, middle-power, and high-power fields, respectively. (PPTX 1063 kb)

## Abbreviations

CAR: Chimeric antigen receptor
DEL: Double-expressing lymphoma
DHL: Double-hit lymphoma
GFP: Green fluorescent protein
PerCP: Peridinin chlorophyll protein complex
