# Supplementary material for: T cells bearing anti-CD19 and/or anti-CD38 chimeric antigen receptors effectively abrogate primary double-hit lymphoma cells
Source: J Hematol Oncol. 2017 Jun 8;10:116. doi: 10.1186/s13045-017-0488-x (PMC5465447; doi:10.1186/s13045-017-0488-x)
Supplement: Supplementary file 1 — Morphology of cells in the specimens on hematoxylin-eosin staining is shown. MYC expression is shown in lymph node specimens from patient 3. LPF, MPF, and HPF denote low-power, middle-power, and high-power fields, respectively. (PPTX 1063 kb) [file 13045_2017_488_MOESM1_ESM.pptx]

## Slide 1
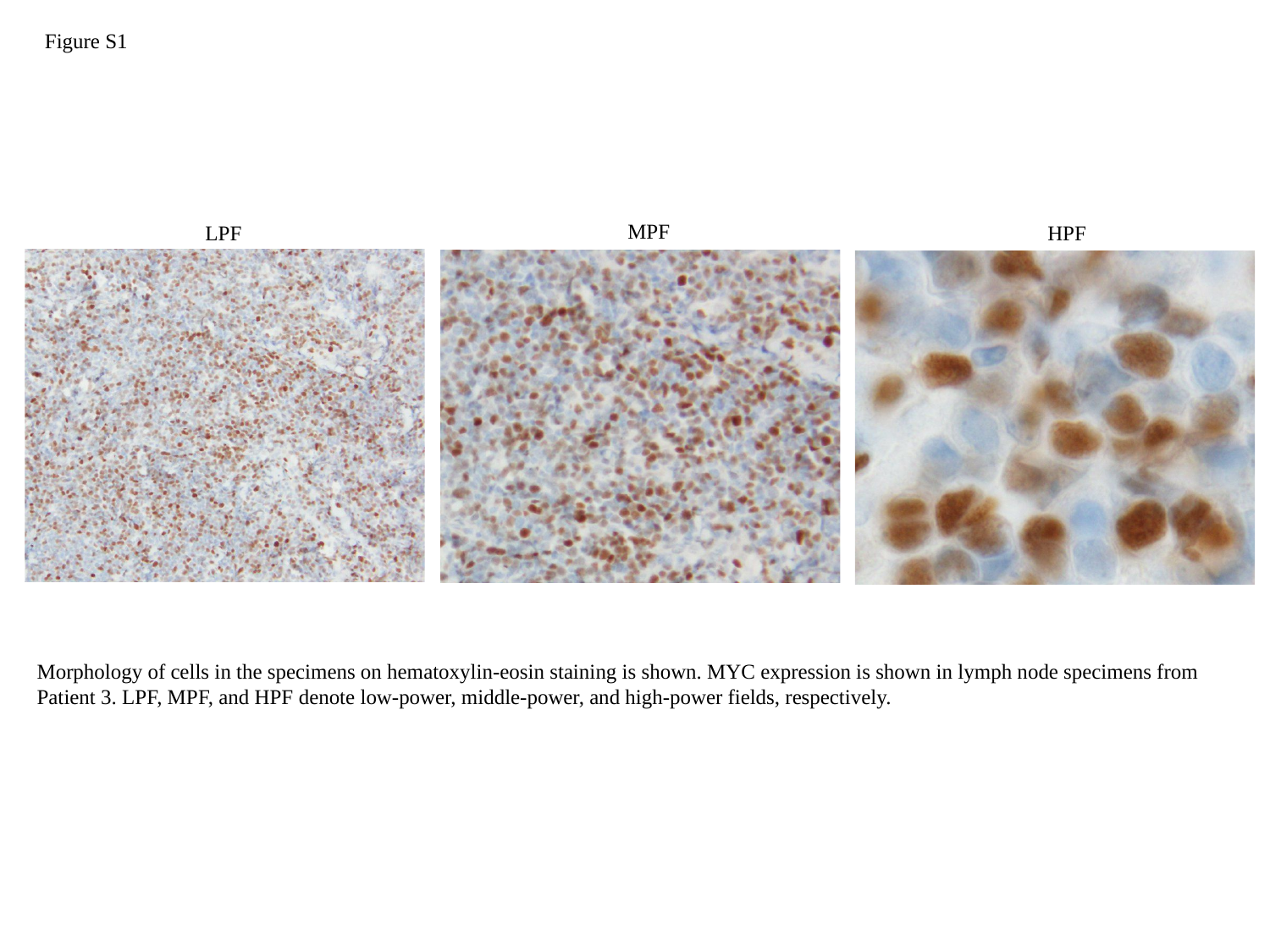

Figure S1
MPF
LPF
HPF
Morphology of cells in the specimens on hematoxylin-eosin staining is shown. MYC expression is shown in lymph node specimens from Patient 3. LPF, MPF, and HPF denote low-power, middle-power, and high-power fields, respectively.
